# Supplementary material for: Multilevel factors drive child exposure to enteric pathogens in animal feces: A qualitative study in northwestern coastal Ecuador
Source: PLOS Glob Public Health. 2024 Sep 18;4(9):e0003604. doi: 10.1371/journal.pgph.0003604 (PMC11410186; doi:10.1371/journal.pgph.0003604)
Supplement: S2 Checklist — (DOCX) [file pgph.0003604.s002.docx]

**Multilevel factors drive child exposure to enteric pathogens in animal feces: A qualitative study in northwestern coastal Ecuador**

**S1. COREQ Checklist**

April M. Ballard^a,b^, Betty Corozo Angulo^c^, Nicholas Laramee^d^, Jayden Pace Gallagher^b^, Regine Haardörfer^e^, Matthew C. Freeman^b^, James Trostle^f^, Joseph N.S. Eisenberg^g^, Gwenyth O. Lee^h^, Karen Levy^i*^, Bethany A. Caruso^b,d,e^

^a^ Department of Population Health Sciences, Georgia State University School of Public Health, Atlanta, Georgia, United States of America

^b^ Gangarosa Department of Environmental Health, Emory University Rollins School of Public Health, Atlanta, Georgia, United States of America

^c^ Universidad Técnica Luis Vargas Torres de Esmeraldas, Esmeraldas, Ecuador

^d^ Hubert Department of Global Health, Emory University Rollins School of Public Health, Atlanta, Georgia, United States of America

^e^ Department of Behavioral, Social, and Health Education Sciences, Emory University Rollins School of Public Health, Atlanta, Georgia, United States of America

^f^ Department of Anthropology, Trinity College, Hartford, Connecticut, United States of America

^g^ Department of Epidemiology, University of Michigan School of Public Health, Ann Arbor, Michigan, United States of America

^h^ Rutgers Global Health Institute and Department of Biostatistics and Epidemiology, Rutgers School of Public Health, Piscataway, New Jersey, United States of America

^i^ Department of Environmental and Occupational Health Sciences, University of Washington School of Public Health, Seattle, Washington, United States of America

*****Email: klevyx@uw.edu

**Consolidated Criteria for Reporting Qualitative research checklist**

| **Item #** | **Checklist Item** | **Location where item is reported** |
| --- | --- | --- |
| **Domain 1: Research team and reflexivity** | | |
| *Personal Characteristics* | | |
| 1 | Author who conducted the interview | Methods: *Data collection*, S2. Reflexivity Statement |
| 2 | Researcher’s credentials | Author list, *Methods: Data collection* |
| 3 | Researcher’s occupation at the time of the study | Author list, S2. Reflexivity Statement |
| 4 | Researcher’s gender | Methods: *Data collection,* S2. Reflexivity Statement |
| 5 | Researcher’s experience and training | Methods: *Data collection*, S2. Reflexivity Statement |
| *Relationship with participants* | | |
| 6 | Relationship established prior to study commencement | S2. Reflexivity Statement |
| 7 | Participants’ knowledge of the interviewer | S2. Reflexivity Statement |
| 8 | Interviewer characteristics reported to participants | S2. Reflexivity Statement |
| **Domain 2: Study design** | | |
| *Theoretical framework* | | |
| 9 | Methodological orientation underpinning the study | Methods: *Data analysis* |
| *Participant selection* | | |
| 10 | Sampling approach | Methods: *Sample and participant selection* |
| 11 | Method of approaching participants | Methods: *Sample and participant selection* |
| 12 | Sample size | Results |
| 13 | Non-participation information | Not applicable (no refusals) |
| *Setting* | | |
| 14 | Setting/location of data collection | Methods: *Data collection* |
| 15 | Presence of non-participants | Not applicable (no others present) |
| 16 | Description of sample | Results |
| *Data Collection* | | |
| 17 | Interview guide provided | S3. Qualitative Data Collection Tools |
| 18 | Repeat interviews | Not applicable (no repeat interviews) |
| 19 | Audio-visual recording | Methods: *Data collection* |
| 20 | Field notes | Methods: *Data collection* |
| 21 | Duration | Methods: *Data collection* |
| 22 | Data saturation | Methods: *Data analysis* |
| 23 | Transcripts returned to participants | Not applicable (transcripts not returned) |
| **Domain 3: Analysis and findings** | | |
| *Data analysis* | | |
| 24 | Number of data coders | Methods: *Data analysis* |
| 25 | Description of the coding tree | S4. Analytic Codes |
| 26 | Derivation of themes | Methods: *Data analysis* |
| 27 | Software | Methods: *Data analysis* |
| 28 | Participant checking | Not applicable (no member checking) |
| *Reporting* | | |
| 29 | Quotations presented and identified | Results |
| 30 | Data and findings consistent | Results |
| 31 | Clarity of major themes | Results, S4. Analytic Codes |
| 32 | Clarify of minor themes | Results, S4. Analytic Codes |
