# Supplementary material for: Multilevel factors drive child exposure to enteric pathogens in animal feces: A qualitative study in northwestern coastal Ecuador
Source: PLOS Glob Public Health. 2024 Sep 18;4(9):e0003604. doi: 10.1371/journal.pgph.0003604 (PMC11410186; doi:10.1371/journal.pgph.0003604)
Supplement: S1 Text — (DOCX) [file pgph.0003604.s003.docx]

**Multilevel factors drive child exposure to enteric pathogens in animal feces: A qualitative study in northwestern coastal Ecuador**

**S2. Reflexivity Statement**

April M. Ballard^a,b^, Betty Corozo Angulo^c^, Nicholas Laramee^d^, Jayden Pace Gallagher^b^, Regine Haardörfer^e^, Matthew C. Freeman^b^, James Trostle^f^, Joseph N.S. Eisenberg^g^, Gwenyth O. Lee^h^, Karen Levy^i*^, Bethany A. Caruso^b,d,e^

^a^ Department of Population Health Sciences, Georgia State University School of Public Health, Atlanta, Georgia, United States of America

^b^ Gangarosa Department of Environmental Health, Emory University Rollins School of Public Health, Atlanta, Georgia, United States of America

^c^ Universidad Técnica Luis Vargas Torres de Esmeraldas, Esmeraldas, Ecuador

^d^ Hubert Department of Global Health, Emory University Rollins School of Public Health, Atlanta, Georgia, United States of America

^e^ Department of Behavioral, Social, and Health Education Sciences, Emory University Rollins School of Public Health, Atlanta, Georgia, United States of America

^f^ Department of Anthropology, Trinity College, Hartford, Connecticut, United States of America

^g^ Department of Epidemiology, University of Michigan School of Public Health, Ann Arbor, Michigan, United States of America

^h^ Rutgers Global Health Institute and Department of Biostatistics and Epidemiology, Rutgers School of Public Health, Piscataway, New Jersey, United States of America

^i^ Department of Environmental and Occupational Health Sciences, University of Washington School of Public Health, Seattle, Washington, United States of America

*****Email: klevyx@uw.edu

**Reflexivity statement**

The training, experience, and characteristics of the research team informed and influenced this qualitative study.

*Prior experience and assumptions*

- **Research background:** Most team members have extensive experience in water, sanitation, and hygiene (WASH), exposure to enteric pathogens in animal feces, and public health (AMB, MCF, JNSE, GOL, KL, BAC). This shaped our study’s design and implementation. Notably, MCF and KL have led reviews on the links between animal-related exposure, enteric pathogens, and diarrheal disease risk. Additionally, AMB, MCF, KL, and BAC have conducted a systematic audit of measures to assess human exposure to enteric pathogens in animal feces.
- **Regional experience:** Team members JNSE and KL have worked together in northern coastal Ecuador for over 16 years, which has included research on child exposure to enteric pathogens and animals. This, along with existing literature and other team members’ experiences, informed our focus on child exposure to enteric pathogens in animal feces as a critical issue.
- **Pilot research:** A pilot study (2019) was conducted by AMB, KL and BAC and guided our study design, emphasizing the importance of observational methods and go-along in-depth interviews to gain key insights into child behavior and environmental conditions.
- **Qualitative expertise and adaptations:** Several members of the team (AMB, BCA, JT, BAC) have experience in qualitative research methods, influencing our study approach. Pilot research highlighted the impracticality of audio recording go-along portions of interviews and the need to adapt our methodological approach to suit the local context. We included observational methods and go-along interviews as a result, which facilitated data triangulation methods to enhance the validity and robustness of our findings.
- **Institutional positionality and power:** All team members except one (BCA) are based in high-income countries, which shaped our perspectives and interpretations. Recognizing our positions as researchers from high-income countries, we were mindful of potential power imbalances and their impact on participant interactions and data interpretation. To minimize the impact of power imbalances, BCA was the only team member with direct participant contact. Other research processes were collaborative to contribute to the validity of our interpretations.
- **Relationship and interactions with participants:** Only BCA had direct face-to-face contact with study participants. With over 10 years of experience in social science and qualitative research, BCA’s background influenced her interactions, questions, and data interpretation. She is from Esmeraldas, the urban community in the study area, and has extensive knowledge about agriculture and animals in the area. This background contributed to her follow-up questions during semi-structured interviews, as well as how she interpreted data. She did not have established relationships with participants prior to the study but she has worked in communities throughout the region over the last 10 years. Prior to beginning interviews for the current study, she introduced herself to participants by telling them her name and her role in the study. Her positionality could have impacted participants’ willingness to talk openly about experiences, which she reflected on throughout data collection, during systematic debriefing sessions with AMB, and in her reflective notes.
- **Cultural sensitivity:** Cultural sensitivity was integral to our research approach and study processes. Local customs and norms were respected, and local collaborators played a crucial role in ensuring the appropriateness of our methods and interactions.

*Language and potential bias*

Language played a critical role in our research. Team members had varying levels of Spanish and English proficiency, which influenced their roles and contributions.

- **Interview guide and short survey:** AMB drafted the interview guide and short survey in English and Spanish, working closely with BCA on Spanish translations. BAC reviewed English versions prior to translations.
- **Interview and analysis collaborations:** AMB worked directly with BCA during data collection and analyses. Communications were both written and verbal to minimize potential misunderstandings or misinterpretations.
- **Transcript and translation:** AMB also oversaw the transcription and translation processes, working closely with one Ecuadorian transcriber and two Ecuadorian translators. Translators were trained on the research topic, interview content, and conducted first-pass transcript reviews. They debriefed with AMB to help ensure the transcripts and translations were high quality.
- **Coding and analysis:** Coding and initial analyses were led by AMB, which NL and JPG collaborated on. NL and JPG did not speak Spanish and relied on English translations for coding and analyses. English translations were stored alongside the original Spanish transcripts, which allowed AMB to interact with the original and translated versions throughout analyses to conduct second-pass transcript reviews and to improve the rigor of interpretations. AMB led cross-checking coding strategies and interpretation of data and debriefing meetings were held throughout coding and initial analyses with AMB, NL, JPG, and BAC.
- **Cross-checking results:** BCA reviewed all draft results to edit, clarify, and expand upon them, as necessary. These drafts were written in English and were translated for her review using an AI translator. As the interviewer, an expert in the area, and a native Ecuadorian, this contributed significantly to the validity of the results. Verbal and written discussions were also had during BAC’s review to ensure understanding and address any mistranslation/misunderstandings due to the use of AI.

*Collaborative knowledge production*

The research team engaged in a collaborative analysis process:

- **Core analysis team:** AMB, BCA, NL, JPG, and BAC worked closely on data analysis, discussing emerging themes, reviewing coded segments, and developing preliminary results.
- **Extended team involvement:** Other team members (RH, MCF, JT, JNSE, GOL, KL) were engaged later in the analysis process, offering broader perspectives and identifying new lines of inquiry. This inclusive approach leveraged the combined insights of those who were closest to the data and those with wider perspectives, ensuring robust engagement despite language barriers.
