# Supplementary material for: Multilevel factors drive child exposure to enteric pathogens in animal feces: A qualitative study in northwestern coastal Ecuador
Source: PLOS Glob Public Health. 2024 Sep 18;4(9):e0003604. doi: 10.1371/journal.pgph.0003604 (PMC11410186; doi:10.1371/journal.pgph.0003604)
Supplement: S1 Data — (DOCX) [file pgph.0003604.s004.docx]

**Multilevel factors drive child exposure to enteric pathogens in animal feces: A qualitative study in northwestern coastal Ecuador**

**S3. Qualitative Data Collection Tools**

April M. Ballard^a,b^, Betty Corozo Angulo^c^, Nicholas Laramee^d^, Jayden Pace Gallagher^b^, Regine Haardörfer^e^, Matthew C. Freeman^b^, James Trostle^f^, Joseph N.S. Eisenberg^g^, Gwenyth O. Lee^h^, Karen Levy^i*^, Bethany A. Caruso^b,d,e^

^a^ Department of Population Health Sciences, Georgia State University School of Public Health, Atlanta, Georgia, United States of America

^b^ Gangarosa Department of Environmental Health, Emory University Rollins School of Public Health, Atlanta, Georgia, United States of America

^c^ Universidad Técnica Luis Vargas Torres de Esmeraldas, Esmeraldas, Ecuador

^d^ Hubert Department of Global Health, Emory University Rollins School of Public Health, Atlanta, Georgia, United States of America

^e^ Department of Behavioral, Social, and Health Education Sciences, Emory University Rollins School of Public Health, Atlanta, Georgia, United States of America

^f^ Department of Anthropology, Trinity College, Hartford, Connecticut, United States of America

^g^ Department of Epidemiology, University of Michigan School of Public Health, Ann Arbor, Michigan, United States of America

^h^ Rutgers Global Health Institute and Department of Biostatistics and Epidemiology, Rutgers School of Public Health, Piscataway, New Jersey, United States of America

^i^ Department of Environmental and Occupational Health Sciences, University of Washington School of Public Health, Seattle, Washington, United States of America

*****Email: klevyx@uw.edu

1. **Short survey**

| **Question** | **Code** | **Response** |
| --- | --- | --- |
| Age of mother | Year | **__ __** |
| Age of child under two years | Months | **__ __** |
| What is the sex of the child? | Male 01  Female 02 | **__ __** |
| How many people live in the household? | Number of people | **__ __** |
| How many children live in the household? | Number of children | **__ __** |
| How many years of education have you completed? | Years | **__ __** |
| What is your ethnicity? | Afro-Ecuadorian  Chachi  Mestizo  Manabí  Other (Make a note): ________________________ | |
| What is the principal material of the floor? | Dirt/sand  Manure  Wooden boards  Palm/bamboo  Parquet/polished wood | Vinyl/asphalt  Ceramic tiles  Cement  Carpet  Other (Make a note):  _____________________ |
| What is the principal material of the roof? | There is no roof  Straw/palm leaves  Grass  Rustic rug  Palm/bamboo  Wooden boards  Cardboard | Metal/Zinc  Wood  Calamine fiber  Ceramic tiles  Cement  Roof tiles  Other (Make a note):  _____________________ |
| What is the principal material of the exterior walls? | There are no walls  Cane/palm/tree trunks  Dirt  Bamboo with mud  Stone with mud  Adobe (uncovered)  Cardboard  Reused wood  Cement | Limestone  Bricks  Cement blocks  Adobe (covered)  Wood planks/shingles  Other (Make a note):  _____________________ |
| What type of bathroom does the household typically use? | Toilet that discharges into sewer system  Toilet that discharges to septic tank  Toilet that unloads to pit latrine  Improved ventilated pit latrine | Pit latrine with slab  Pit latrine without slab  Latrine/composting toilet  Plastic bucket used as a toilet  Hanging toilet/latrine  No facility/bush/field  Other (Make a note):  _____________________ |
| What source of water do you use to prepare fluids for your child? | Tube well  Protected well  Unprotected well  Protected spring  Unprotected spring  Surface water- river  Surface water- stream | Piped water connection  Public tap  Small tank truck/drum  Rainwater  Bottled water  Other (Make a note): _____________________ |
| What animals do you have and how many of each? | Production chickens _____  Free-range chickens _____  Ducks _____  Turkeys _____  Guinea pigs _____  Dogs _____  Pigs _____  Cows _____ | Horses/mules/donkeys _____  Sheep/goats _____  Cats _____  Bush rats _____  Parrots _____  Other (Make a note):  _____________________ |

1. **Semi-structured in-depth interview guide**

*For interviewer to read: We would like to learn about the interactions and contact that you and your child have with people, animals, and the environment. The interview will focus on a description of a typical day and your daily activities, and we will ask you to show us and comment on the activities you do. The interview will be more like a conversation. There are no right or wrong answers, we just want to know your opinions and experiences.*

1. We will start at the beginning of a typical or normal day. Thinking about when you wake up, can you describe what you do in the mornings? Where are you and who is there?

- What does your child do in the morning?
  - Where are they and who are they with?
  - Who else is present and what are those people doing?
- Do any animals enter inside [insert location where mother and child report] in the mornings? If so, what types and who do they belong to?
  - Where are the animals exactly and what are they doing?
  - What are people doing with the animals (e.g., having contact, feeding them, playing with them)?
  - What type of contact does your child have with the animals?
  - Can you describe what type of interactions or contact your child under two has with the animals?
  - Who do the animals belong to?
  - How long are they present and how often are they present?
  - Do animals defecate or are animal feces present inside [insert location where mother and child report] in the mornings?
    - How often does this occur?
    - What happens when this occurs?
    - Are there differences in what happens based on the type of animal feces? If so, why, and what are the differences?
- Are there any animals outside near [insert location where mother and child report] in the mornings? If so, what types and who do they belong to?
  - Where are the animals exactly and what are they doing?
  - What are people doing with the animals (e.g., having contact, feeding them, playing with them)?
  - Can you describe what type of interactions or contact your child under two has with the animals?
  - Who do the animals belong to?
  - How long are they present and how often are they present?
  - Do animals defecate or are animal feces outside [insert location where mother and child report] in the mornings?
    - How often does this occur?
    - What happens when this occurs?
    - Are there differences by the type of animal feces?
    - If so, why, and what are the differences?
    - Do animal feces get brought inside the house or other location where you spend the mornings (e.g., through shoes, toys, or in any other way)?
- Can you show me where these activities occur?

1. Okay so you and your child do **_______** in the mornings. From what and until what time? What do you do after that? Where do you spend most afternoons?

- What about your child during that time? Do they go with you? Do they go somewhere else?
  - Where are they and who are they with?
  - Who else is present and what are those people doing?
- Are there any animals inside [insert location where mother and child report] in the afternoons? If so, what types and who do they belong to?
  - Where are the animals exactly and what are they doing?
  - What are people doing with the animals (e.g., having contact, feeding them, playing with them)?
  - Can you describe what type of interactions or contact your child under two has with the animals?
  - Who do the animals belong to?
  - How long are they present and how often are they present?
  - Do animals defecate or are animal feces present inside [insert location where mother and child report] in the afternoons?
    - How often does this occur?
    - What happens when this occurs?
    - Are there differences by the type of animal feces?
    - If so, why, and what are the differences?
- Are there any animals outside near [insert location where mother and child report] in the afternoons? If so, what types and who do they belong to?
  - Where are the animals exactly and what are they doing?
  - What are people doing with the animals (e.g., having contact, feeding them, playing with them)?
  - Who do the animals belong to?
  - How long are they present and how often are they present?
  - Do animals defecate or are animal feces outside [insert location where mother and child report] in the afternoons?
    - How often does this occur?
    - What happens when this occurs?
    - Are there differences by the type of animal feces?
    - If so, why, and what are the differences?
    - Do animal feces get brought inside the house or other location where you spend the afternoons (e.g., through shoes, toys, or in any other way)?
- Can you show me where these activities occur?

1. Great, now imagine the afternoon is over. Normally, what do you do next?

- What about your child during that time? Do they go with you? Do they go somewhere else?
  - Where are they and who are they with?
  - Who else is present and what are those people doing?
- Are there other people there? What are they doing?
- Are there any animals inside [insert location where mother and child report] in the evenings? If so, what types and who do they belong to?
  - Where are the animals exactly and what are they doing?
  - What are people doing with the animals (e.g., having contact, feeding them, playing with them)?
  - Can you describe what type of interactions or contact your child under two has with the animals?
  - Who do the animals belong to?
  - How long are they present and how often are they present?
  - Do animals defecate or are animal feces present inside [insert location where mother and child report] in the evenings?
    - How often does this occur?
    - What happens when this occurs?
    - Are there differences by the type of animal feces?
    - If so, why, and what are the differences?
- Are there any animals outside near [insert location where mother and child report] in the evenings? If so, what types and who do they belong to?
  - Where are the animals exactly and what are they doing?
  - What are people doing with the animals (e.g., having contact, feeding them, playing with them)?
  - Who do the animals belong to?
  - How long are they present and how often are they present?
  - Do animals defecate or are animal feces outside [insert location where mother and child report] in the evenings?
    - How often does this occur?
    - What happens when this occurs?
    - Are there differences by the type of animal feces?
    - If so, why, and what are the differences?
    - Do animal feces get brought inside the house or other location where you spend the evenings (e.g., through shoes, toys, or in any other way)?
- Can you show me where these activities occur?

*Thank you. Now I’d like to talk about animals in your community and any animals that you own.*

1. In your community, what types of animals do people have?

- What types of animals are most common?
- What motivates people to have these animals (e.g., consumption, income, companionship, security)?
- What are the benefits of having animals?

1. (*If the household owns animals, ask this question and the probes for each type of animal*) Why do you have animals?

- What are the benefits of having the animal?
- Have you always had these animals, or has it changed over time? If so, why?
  - What influences you to not have animals (e.g., are there certain times of year or seasons that impact having animals)?
- How did your family decide to have animals? Who was involved in the decision?
  - What was the process for deciding?

1. Have you had animals in the past? If so, what type(s)?

- What was done to care for the animals and who decided how to care for them?
- Were the animals allowed inside? If so, who decided if the animals could be inside?
  - Were there differences by the type of animals? Why or why not?
- Could your child have contact with the animals? If so, which ones and why?
  - Who decided about the contact?
- Why do you not have those animals now?
  - How was the decision not to have animals made, and by who?
    - What was the process for deciding?

1. (*If the household owns animals, ask this question and the probes for each type of animal*) Who is responsible for the animals and why?

- What is done to care for the animals and who decides how to care for them?
  - Does the rainy season impact how you care for the animals? If so, how and why?
  - Does the rainy season impact your child? For example, does it affect how they play or how close they are to animals?
- Are the animals allowed inside? If so, who decides if the animals can be inside?
  - Are there differences by the type of animals? Why or why not?
- Can your child have contact with any animals? If so, which ones and why?
  - Who decides about this contact?

1. Can you show me where your animals live and how you care for them?
