# Supplementary material for: Multilevel factors drive child exposure to enteric pathogens in animal feces: A qualitative study in northwestern coastal Ecuador
Source: PLOS Glob Public Health. 2024 Sep 18;4(9):e0003604. doi: 10.1371/journal.pgph.0003604 (PMC11410186; doi:10.1371/journal.pgph.0003604)
Supplement: S2 Data — (DOCX) [file pgph.0003604.s005.docx]

**Multilevel factors drive child exposure to enteric pathogens in animal feces: A qualitative study in northwestern coastal Ecuador**

**S4. Analytic Codes**

April M. Ballard^a,b^, Betty Corozo Angulo^c^, Nicholas Laramee^d^, Jayden Pace Gallagher^b^, Regine Haardörfer^e^, Matthew C. Freeman^b^, James Trostle^f^, Joseph N.S. Eisenberg^g^, Gwenyth O. Lee^h^, Karen Levy^i*^, Bethany A. Caruso^b,d,e^

^a^ Department of Population Health Sciences, Georgia State University School of Public Health, Atlanta, Georgia, United States of America

^b^ Gangarosa Department of Environmental Health, Emory University Rollins School of Public Health, Atlanta, Georgia, United States of America

^c^ Universidad Técnica Luis Vargas Torres de Esmeraldas, Esmeraldas, Ecuador

^d^ Hubert Department of Global Health, Emory University Rollins School of Public Health, Atlanta, Georgia, United States of America

^e^ Department of Behavioral, Social, and Health Education Sciences, Emory University Rollins School of Public Health, Atlanta, Georgia, United States of America

^f^ Department of Anthropology, Trinity College, Hartford, Connecticut, United States of America

^g^ Department of Epidemiology, University of Michigan School of Public Health, Ann Arbor, Michigan, United States of America

^h^ Rutgers Global Health Institute and Department of Biostatistics and Epidemiology, Rutgers School of Public Health, Piscataway, New Jersey, United States of America

^i^ Department of Environmental and Occupational Health Sciences, University of Washington School of Public Health, Seattle, Washington, United States of America

*****Email: klevyx@uw.edu

**Themes and sub-themes with analytic codes**

| **Theme** | **Definition** | **Sub-theme(s)** | **Parent codes (child codes)** |
| --- | --- | --- | --- |
| Child behaviors | Behaviors that may lead to exposure to enteric pathogens inside and outside of child households and in other locations where animals and animal feces were present | 1. Playing on floor, soil, or sand 2. Contact with animals 3. Mouthing soil and sand in environment or on objects | - Human behavior (fomites, direct child contact with animals, child exposure to feces) - Animals (presence of animals inside of household, outside near household, inside non-household location, outside non-household location) - Environment (fecal contamination of environment inside home, outside but near the home, inside another space, outside another space) |
| Child environment | Close surroundings and daily conditions in which children live, have direct contact with, and impact their proximity and potential exposure to animal feces | 1. Others who have contact with animals and animal feces 2. Built environment features | - Human behavior (other family member contact with animals, other family member contact with animal feces, others caring for child) - Other exposure factors (household characteristics and behaviors that influence exposure) |
| Community norms | Sets of behaviors or practices that are widely accepted and expected within a community that influence child proximity to animals and animal feces | 1. Animal management and husbandry 2. Animal feces management | - Human behavior (other family member contact with animals, other family member contact with animal feces) - Animals (animal living conditions, animal husbandry practices, animal health, presence of animals inside of household, outside near household, inside non-household location, outside non-household location) - Environment (fecal contamination of environment inside home, outside but near the home, inside another space, outside another space) - Animal ownership and decision-making (reasons and benefits for, variability of, and decision-making about animal ownership) |
| Natural environmental conditions | Atmospheric characteristics of a geographic area based on its climate and seasonal weather patterns that influence child proximity to animals and animal feces | 1. Extreme weather events | - Other exposure factors (household characteristics and behaviors that influence exposure) - Animal ownership and decision-making (variability of animal ownership, effect of seasonality and weather, decision-making about animal ownership) |
