# Supplementary material for: Multilevel factors drive child exposure to enteric pathogens in animal feces: A qualitative study in northwestern coastal Ecuador
Source: PLOS Glob Public Health. 2024 Sep 18;4(9):e0003604. doi: 10.1371/journal.pgph.0003604 (PMC11410186; doi:10.1371/journal.pgph.0003604)
Supplement: Attachment — Submitted filename: Qual R&R response to reviewers.docx [file pgph.0003604.s006.docx]

**PLOS Global Public Health – Response to reviewers**

We thank the journal and reviewers for their comments. The suggestions were valuable, and the revisions made have strengthened this publication. We provide a point-by-point response to reviewer’s comments and a revised version of our manuscript. The lines that we reference in our response are specific to the document with tracked changes.

| Comments/requested revisions | Authors’ response and additions /edits to manuscript (as applicable) |
| --- | --- |
| **Journal requirements** | |
| J1. Please include a complete copy of PLOS’ questionnaire on inclusivity in global research in your revised manuscript. Our policy for research in this area aims to improve transparency in the reporting of research performed outside of researchers’ own country or community. The policy applies to researchers who have travelled to a different country to conduct research, research with Indigenous populations or their lands, and research on cultural artefacts. The questionnaire can also be requested at the journal’s discretion for any other submissions, even if these conditions are not met. Please find more information on the policy and a link to download a blank copy of the questionnaire here: https://journals.plos.org/globalpublichealth/s/best-practices-in-research-reporting. Please upload a completed version of your questionnaire as Supporting Information when you resubmit your manuscript.” | We have completed and included the questionnaire. |
| J2. Please amend your detailed Financial Disclosure statement. This is published with the article. It must therefore be completed in full sentences and contain the exact wording you wish to be published.   a. State the initials, alongside each funding source, of each author to receive each grant. b. If any authors received a salary from any of your funders, please state which authors and which funders.   If you did not receive any funding for this study, please simply state: “The authors received no specific funding for this work. | We have updated the detailed Financial Disclosure statement. |
| **Reviewers’ comments** | |
| R2.1. This article authored by Ballard et al., discusses an interesting topic on the risks of children aged 6–18 months being exposed to animal faeces, which can lead to exposure to harmful enteric pathogens. It emphasises the importance of combining animal and child faeces management programmes to decrease the risk of exposure to these pathogens, which are linked to various health issues commonly seen in childhood. The article is impressive for its rigorous methods and its substantive contribution to the existing body of knowledge in this field. However, there are some specific areas for improvement. | We appreciate the reviewer’s comments and attention to the rigor of our approach. |
| R2.2. Abstract  Lines 13–14: The abstract should emphasise the observations made in the study rather than compare them to previous findings. The discussion section would be a more appropriate place for that. | The referenced lines, along with Lines 15-20, represent the conclusion section of the abstract, which is used to express the primary take-home message and the implications or importance of the findings for the field. The reference to unsafe child feces management practices is included to highlight the practical implications of the findings, which is appropriate for the conclusion section. We therefore did not make any changes. |
| R2.3. Introduction   a. The introduction is perfectly put- together. However, the authors should consider including statements about the prevalence of infectious and diarrheal diseases in the region where the study took place. b. Line 37: The authors should specify which enteric pathogens are being considered here. | We thank the reviewer for these important points.   a. Studies have estimated the 2-week prevalence of diarrhea among children under age five to be around 9%, and of enteropathogenic *E. coli* infections to be around 25%. We have added this to the manuscript (Lines 52-54). b. We have added a few examples to the sentence on Line 37 to help clarify.   “…many enteric pathogens capable of infecting humans are found in animal feces (e.g., *Campylobacter* spp., *Cryptosporidium* spp., enteropathogenic *E. coli*).” This study is broadly focused on enteric pathogens in animal feces, which may include 15 or more pathogens outlined in Delahoy et al. Because our goal is to qualitatively explore opportunities for and factors that influence child exposure to pathogens in animal feces, a specific list of enteric pathogens is outside of the current study’s scope. |
| R.2.4. Sample And Participant Selection   a. This section should be edited to focus on the sample selection and the criteria used for inclusion and exclusion. Additionally, the results presented in Table 1 should be relocated to the results section. b. The abstract indicates that the study included children aged 6–18 months, yet the methods section highlights that children aged 6–10 months were excluded. May the authors clarify to ensure that the age ranges are clearly defined? The final sample only included those aged 10–18 months; therefore, the study is considered to be focused on that age group. | a. We have moved Table 1 to the Results section to make the section clearer. The referenced section discusses the sample selection and eligibility criteria and includes justification for each, as written. b. We adjusted the language in the abstract to clarify that interviews were **conducted** with mothers of children aged 10-18 months. As stated in the Methods, “mothers were eligible if their child in the cohort was between 6-18 months old. This age range was selected because children become more mobile and active during this time, making them particularly susceptible to environmental exposures” (Lines 89-91). Our final sample did not include children between 6-10 months old because few children that age were enrolled in the cohort at the time of recruitment due to a pause in the study at the beginning of the COVID-19 pandemic (Lines 187-189). This information is already described in the manuscript. |
| R.2.5. Data Collection  Line 126: There is a need to provide sufficient detail on how the translation process was conducted to ensure reliability. | We appreciate the reviewer’s attention to detail and have added the following information to the manuscript (Lines 134-141): “Verbatim translations were performed by two Ecuadorians. To standardize transcript formatting and obtain quality translations, we trained the translators on the research topic, interview content, first-pass transcript reviewing while translating, and goal of achieving meaning equivalence. Author AMB also debriefed with translators after each of the initial five translations were completed and checked translations for accuracy. English translations were stored alongside the original Spanish transcripts, which allowed us to interact with the original and translated versions throughout analyses to improve the rigor of our interpretations.” |
| R.2.6. Strengths and Limitations  The authors should restructure the section to present the strengths first, followed by the limitations. In its current state, the section is somewhat cluttered. Sample selection limitations should be emphasized, as the exclusion of those aged 6 - 10 months surely would have an impact on the interpretation of the study results. | We have adjusted the ‘strengths and limitations’ section for readability and added information about our final sample only including children 10-18 months old. |
| R.3.1. This article brings a new insight into child exposure to enteric pathogens through animals' feces. This should be taken into account in the effort for child protection against pathogen. As this is a qualitative study, my main recommendation to the author is to add more verbatims from mothers' opinion to support the summaries written under different level of exposure aspects of the study. Below are my specific comments on this manuscript. | We appreciate the reviewer’s comments and address the specific recommendations below. |
| R.3.2. Line 103. Table 1. Under child characteristics  Sex – male : It is supposed that figures pertaining to Sex-male are absolute frequency. The authors need to add precision in the first column about figures between brackets (i.e. (48) ?) | The values in the row with ‘Sex – male’ in Table 1 do indeed correspond with frequency. We have added ‘(n(%))’ directly after the label, as is done for all other values in the table. |
| R.3.3. To make the table 1 clearer and more readable, there a need to insert a title line Under Household characteristics to introduce the size. Size (mean [range]) as it is presented for other characteristics. This will avoid mention of (mean [range]) for each line. Size (mean [range]) Household size 5 (3-13) …# of children 3 (1-6)… | We have made this adjustment in Table 1 to improve readability. |
| R.3.4.   a. “Owns animals (n(%))“ can be replaced by “Pet ownership (n(%))” b. Figures on the same line with the variable name should be written below the variable name by inserting a line with a category that can be stated as   “All/overall” 32 (55) 11 (61) 10 (50) 7 (50) 4 (67) | We appreciate this point from the reviewer.   a. Pet ownership does not accurately describe all animals included (e.g., chickens) in Table 1. We therefore did not change ‘Owns animals’ to ‘Pet ownership.’ b. We have added a line under ‘Owns animals’ for ‘Any’ to present overall ownership frequencies on a separate line. |
| R.3.5. There is a need to add more verbatims to make mother talk about what is summarized by the authors:   a. At the end of the section entitled “Child environment”, it can be inserted starting on line 217. b. In “Others’ contact with animals and animal feces”, a verbatim can be added at line 222 after the full stop. The sentence starting with “However,” can be moved after this verbatim. c. The sentence on line 267 to 269 needs to be supported by verbatims from mothers’ opinion on this statement. d. The sentence on lines 289 and 290 needs to be supported by verbatim from mothers. It can be inserted below line 293. e. Below line 299, there is a need to illustrate how mothers mentioned community norms and how do community norms influence child exposure to animals’ feces. f. One or two verbatims should be added to support authors last summary on “animal management and husbandry”. They can be inserted below the line 334. | We have added six illustrative quotes to complement the 13 that were previously provided, as the use of verbatims is one approach for validating qualitative results, used as a way of demonstrating that the findings are indeed evident in the data in the way described. Author BCA, who conducted all interviews, also contributed to and cross-checked the analyses and results, which is a well-established and equally important approach to validate qualitative results (described in S1 File: *Reflexivity statement*).   a. Some qualitative results that are equally valid and well-grounded may have no clear quotation to illustrate the concept itself. This is especially true when a particular result collectively represents a broader concept in the data, which is the case for the referenced key theme ‘Child environment.’ As such, we did not add a quote at the end of section. However, many quotes are provided to support the ‘Child environment’ sub-themes, which were used to develop the broader ‘Child environment’ theme. b. We added the following quote to support the statement about some mothers reporting intense contact with animals: “I keep [the dogs and cats] clean so that they don’t get fleas, ticks, or any of that. I wipe them down over there. They sleep in a dry place, and I keep the outside area where they poop clean. If I ever see a tick, they get an injection, or I wash them with chemicals” (Lines 255-258). c. The following quote was added: “I don’t allow [animals to get near the house] because they get used to it. There is a dog that knew that I put food out sometimes so as not to throw it away, but I don’t give it to him anymore because they get used to it” (Lines 306-309). d. We added the following quote to support the referenced paragraph: “We always clean at my mother’s place because the children play outside a lot. As I say, I go to my mother’s house every day. Sometimes we sweep together… [the animal feces] are picked up, but I would say yes [feces are brought inside] because sometimes they step on it without noticing” (Lines 334-338). e. As stated above, some qualitative results that are equally valid and well-grounded may have no clear quotation to illustrate the concept itself, which is the case for the broader ‘Community norms’ theme. As such, we did not add a quote at the end of the section. However, we have added two quotes to the ‘Animal management and husbandry’ subtheme section, which were used alongside the ‘Animal feces management’ subtheme to develop the broader ‘Community norms’ theme. f. We did not add quotes to the referenced subsection because we have already provided one in-text quote and six additional quotes in Table 2, providing substantial verbatim evidence to support our results. Additionally, too many quotations can distract from the central message of the results. |
| R.4.1. I would like to thank the authors for their manuscript. Understanding the factors of child exposure to enteric pathogens is a critical topic to help reduce the disease burden of enteric pathogens. This study which focuses specifically on communities in Ecuador can provide important lessons for countries with similar settings. The authors have written a good Introduction which helps set the stage for the study. However, the methodology requires additional information to enhance transparency and reproducibility, and the authors need to re-assess how to present the results section so it answers the objectives of the study. Although this is a good first draft, I have additional suggestions, comments, and edits to enhance the manuscript. Please refer to my comments below for a full review. | We appreciate the reviewer’s comments and agree that additional information could enhance transparency and reproducibility. We respond to the specific recommendations below. |
| R.4.2. Introduction:   a. Line 23: “are the fifth leading cause of death in children under age five”. Please clarify whether this is globally, nationally or regionally. b. Lines 51-57: The authors should consider providing 1-2 lines on their rationale for conducting the study in Ecuador (example - burden of enteric infections etc.) | a. Enteric infections and diarrheal diseases are the fifth leading cause of death in children under age five globally. We have added this point of clarification to the introduction. b. This study was conducted among children in northwestern coastal Ecuador, a high enteric pathogen transmission setting. Previous studies have estimated the two-week prevalence of diarrhea among children under age five to be about 9% and of enteropathogenic *E. coli* infections to be around 25%. This has been added to the introduction (Lines 43-55). |
| R.4.3. I would recommend authors follow Consolidated Criteria for Reporting Qualitative Research (COREQ) checklist for reporting qualitative research. While the authors have provided details on their methodological approach, some additional details are required. The authors should explicitly mention how the study adheres to the COREQ guidelines for reporting qualitative research. COREQ includes specific reporting criteria related to research team & reflexivity, participant selection, data collection, and data analysis, which would enhance the transparency and quality of the study. Please follow the sub-headings reported in COREQ for updating the methods section. | We thank the reviewer for this suggestion, which has enhanced the transparency of our approach and findings. We have ensured that our manuscript follows the COREQ standards, and we have added a table with the COREQ checklist and the location(s) where each item is reported in S1 File. We have also included a reflexivity statement in this supplementary file to reflect on how the training, experience, and characteristics of our research team informed and influenced this qualitative study. |
| R.4.4.   a. Line 63: Would recommend fully defining ECoMiD (Enteropatogenos, Crecimiento, Microbioma y Diarrea.) Please also mention that is a prospective cohort study. b. Line 66-70: It would be great if the authors could include a geographic map of the seven study locations. c. Table 1: Row Education – Please clarify what the unit of measure is? Years? Grade? d. Line 107-108: Under research team and reflexivity, what was the positionality of BCA and what impacts might this have had on the findings? A lot of this information can be detailed in Supplementary Methods. e. Lines 108-110- Please provide the semi-structured Interview Guide in the Supplementary Methods for transparency and reproducibility. f. Lines 116-118: I’m assuming all the information on basic demographics, household characteristics etc. is what is shown in Table 1. Would be helpful for authors to provide the survey questionnaire in the Supplementary File as well. g. Lines 118-120: Would recommend moving this to Data Analysis section. h. Lines 121-128: Please indicate how the transcripts were generated and how accurate was this transcription process? Was this done using software or manually? How were transcripts checked for validity, reliability, and completeness? i. Lines 132-134: Using a Table, please provide the themes/sub-themes and their description that emerged from the thematic content analysis. Please include this codebook in Supplementary Files for transparency and reproducibility. | a. We have adjusted the text to state “These mothers were participating in the Enteropatógenos, Crecimiento, Microbioma, y Diarrhea (ECoMiD) study, a prospective cohort study in which mother-child dyads are followed from pregnancy through the critical first 24 months of life…” b. We have added a map that shows the location of the seven study sites (Fig 1, Line 88). c. The education row refers to years. We have added this to Table 1 to clarify. d. We have included a reflexivity statement in S1 File to reflect on how the training, experience, and characteristics of our research team informed and influenced this qualitative study, including for BCA. e. We have provided the semi-structured interview guide in a Supplementary File (S1 File) for readers. f. The reviewer’s assumption is correct. We have provided the short survey that captured basic information reported in Table 1 in a Supplementary File (S1 File) to make this clearer for readers. g. We have moved Table 1 to the Results section to make the section clearer. h. We have added the following information to the manuscript (Lines 134-141): Transcripts were transcribed and de-identified by an Ecuadorian, and then translated from Spanish to English by two other Ecuadorians. To standardize transcript formatting and obtain quality transcriptions and translations, we trained the translators on the research topic, interview content, conducting first-pass transcript reviewing while translating, and the goal of achieving meaning equivalence. Author AMB debriefed with translators after each of the initial five translations were completed and checked translations for accuracy. English translations were stored alongside the original Spanish transcripts, which allowed us to interact with the original and translated versions throughout analyses to conduct second-pass transcript reviews and to improve the rigor of our analyses. i. We have created a supplemental table that includes the themes, subthemes, and analytic codes from our thematic analysis. This is included in S1 File. |
| R.4.5. Results  In the introduction, the authors define their objectives as “explore the interrelated community, household, and individual child factors that influence exposure. We explore opportunities for and factors that influence exposure across multiple communities along an urban-rural gradient with a range of conditions to increase the applicability to other LMICs. We also examine how household animal ownership influences exposure opportunities, which can provide important insights for potential mitigation strategies.”  I would strongly recommend that authors re-arrange their results section to follow their objectives in sequential order for readability.  For example, I don’t see clear results on the opportunities for and factors that influence exposure by rural and urban settings, as the authors have mentioned in their introductory objectives. Are there any stark differences seen in results in Table 2. Community-level animal feces removal and disposal practices and factors that influence their use, when stratified by urban and rural settings?  While the results are very interesting and rich, it’s hard to tease out results for each of the objectives in the study. I would suggest that the authors re-consider and re-arrange the results on how to explain their findings, so it tells a cohesive story. In its current form, the results feel disjointed and not linked to the three study objectives. | We thank the reviewer for the comments about the results and for the opportunity to improve the clarity of the manuscript. This study had one main objective: to explore opportunities for and factors that influence exposure at multiple levels (e.g., community, household, individual). Our objective was **not** to compare opportunities for and factors that influence exposure by community type, but instead it was to explore them for the entire sample. Multiple communities along an urban-rural gradient with a range of conditions were included to increase the applicability of our findings. To clarify this, we have made the following edits:  “We explore opportunities for and factors that influence child exposure at multiple levels (e.g., community, household, individual), including multiple communities along an urban-rural gradient with a range of conditions to increase the applicability to other LMICs…” (Lines 56-59).  Examining how household animal ownership influences opportunities and factors is a sub-objective in this study. Findings on how animal ownership influences exposure opportunities and factors are therefore integrated throughout the results, which we deem appropriate given that the sub-objective is a specific aspect that we investigated within the main framework of our study. We therefore did not re-arrange the results but did ensure that the objective and sub-objective are clearer in the introduction. |
| R.4.6. The resolution on both figures needs to be higher. | We agree with the reviewer. The poor resolution is due to the editorial manager submission system and is not with figures themselves. |
| R.4.7. Discussion:   a. I would recommend authors discussing the findings of related to other papers in the literature, including quantitative studies.   1. Baker KK, Mumma JAO, Simiyu S, Sewell D, Tsai K, Anderson JD, MacDougall A, Dreibelbis R, Cumming O. Environmental and behavioural exposure pathways associated with diarrhoea and enteric pathogen detection in 5-month-old, periurban Kenyan infants: a cross-sectional study. BMJ Open. 2022 Oct 31;12(10):e059878.  2. Knee J, Sumner T, Adriano Z, Berendes D, de Bruijn E, Schmidt WP, Nalá R, Cumming O, Brown J. Risk factors for childhood enteric infection in urban Maputo, Mozambique: A cross-sectional study. PLoS Negl Trop Dis. 2018 Nov 12;12(11):e0006956.  3. Chard AN, Levy K, Baker KK, Tsai K, Chang HH, Thongpaseuth V, Sistrunk JR, Freeman MC. Environmental and spatial determinants of enteric pathogen infection in rural Lao People's Democratic Republic: A cross-sectional study. PLoS Negl Trop Dis. 2020 Apr 8;14(4):e0008180.   b. Another limitation of the study could be the bias introduced by interviewers during the key informant interviews due to probing or leading questions. Please address this in the manuscript as well. | a. Our findings add to existing qualitative and quantitative research that demonstrates that child household and play spaces can be contaminated with animal feces and associated enteric pathogens when free-range animals are present, and that proximity to animals and such contaminants increases the risk of enteric infections among children. The papers provided by the reviewer are among this existing research and have been added as references. We did not make adjustments to the discussion beyond this as it was unclear what specific recommendation the reviewer was making. As written, we position our findings among 19 existing studies (including the three additional ones recommended by the reviewer), which appropriately addresses the reviewer’s recommendation as we understand it. b. We have added information about BCA’s positionality, as well as other study team members, to a reflexivity statement in S1 File. The identity and positionality of BCA, as well as other research team members, influenced all aspects of our study, including the research question, study design, data collection, and data analysis. This influence is inherent in all research and is not necessarily a limitation, but rather something that should be disclosed for readers to consider alongside results. BCA is from Esmeraldas, the urban community in the study area, and has extensive knowledge about agriculture and animals in the area. This background contributed to her follow-up questions during semi-structured interviews, as well as how she interpreted data. Overall, her positionality could have impacted participants’ willingness to talk openly about experiences, which she reflected on throughout data collection, during systematic debriefing sessions with AMB, and in her reflective notes. |
| R.5.1. General comment: The authors have presented an interesting, well-written manuscript that emphasizes an important topic in child health with a good flow. | We thank the reviewer for their reflections. |
| R.5.2. The manuscript should include country-specific information on diarrhea caused by enteric pathogens in children under five. Including this information will inform the readers of the prevalence of the problem in Ecuador. It's important to include data from both human and animal health sources to provide a comprehensive understanding of the issue. | This study was conducted among children in northwestern coastal Ecuador, a high enteric pathogen transmission setting. Previous studies have estimated the two-week prevalence of diarrhea among children under age five to be about 9% and of enteropathogenic *E. coli* infections to be around 25%. This has been added to the introduction (Lines 43-55). Animal health is outside the scope of this study. |
| R.5.3. The first question that came to mind while reading was why the age restriction was 6-18 months. It is noted that this study was part of the ECoMid study, which had children within that age range. However, unless the reader goes to the reference for the ECoMid study protocol, it would be useful for the author to expand a bit on this selection of age and why not 5 years and under | Mothers recruited for this study were participating in the ECoMiD study, a prospective cohort study in which mother-child dyads are followed from pregnancy through 24 months. Mothers were eligible if their child in the cohort was between 6-18 months old. This age range was selected because children become more mobile and active during this time, making them particularly susceptible to environmental exposures. This information is already included in the Methods section (Lines 67-69; Lines 94-96). We did however add this phrase to emphasize why ECoMiD focused on children under two years: “through the critical first 24 months of life…” (Lines 68-69). |
| R.5.4. Was there a question that asked to the mothers or guardians whether the child had experienced episodes of diarrhea in the recent past? | Information on child diarrhea was not collected, as our study was focused on if, why, how, and to what extent child may be exposed to enteric pathogens in animal feces using qualitative methods. |
| R.5.5. Table 1: Maternal characteristics, Education (mean [range]): It is not clear what those figures represent. | The education row refers to years. We have added this to Table 1 to clarify. |
| R5.6. Attaching the questionnaires (English version) as supplementary information would be helpful to understand how the data was collected | We have provided the semi-structured interview guide and short survey in a Supplementary File (S1 File). |
