# Supplementary material for: Multilevel factors drive child exposure to enteric pathogens in animal feces: A qualitative study in northwestern coastal Ecuador
Source: PLOS Glob Public Health. 2024 Sep 18;4(9):e0003604. doi: 10.1371/journal.pgph.0003604 (PMC11410186; doi:10.1371/journal.pgph.0003604)
Supplement: Attachment — Submitted filename: Qual R&R response to reviewers_2.docx [file pgph.0003604.s007.docx]

**PLOS Global Public Health – Response to reviewers**

We thank the journal and reviewers for their comments. The suggestions were valuable, and the revisions made have strengthened this publication. We provide a point-by-point response to reviewer’s comments and a revised version of our manuscript. The lines that we reference in our response are specific to the document with tracked changes.

| Comments/requested revisions | Authors’ response and additions /edits to manuscript (as applicable) |
| --- | --- |
| **Journal requirements** | |
| J1. Please include a complete copy of PLOS’ questionnaire on inclusivity in global research in your revised manuscript. Our policy for research in this area aims to improve transparency in the reporting of research performed outside of researchers’ own country or community. The policy applies to researchers who have travelled to a different country to conduct research, research with Indigenous populations or their lands, and research on cultural artefacts. The questionnaire can also be requested at the journal’s discretion for any other submissions, even if these conditions are not met. Please find more information on the policy and a link to download a blank copy of the questionnaire here: https://journals.plos.org/globalpublichealth/s/best-practices-in-research-reporting. Please upload a completed version of your questionnaire as Supporting Information when you resubmit your manuscript. | We have completed and included the questionnaire. |
| J2. Please review your reference list to ensure that it is complete and correct. If you have cited papers that have been retracted, please include the rationale for doing so in the manuscript text or remove these references and replace them with relevant current references. Any changes to the reference list should be mentioned in the rebuttal letter that accompanies your revised manuscript. If you need to cite a retracted article, indicate the article’s retracted status in the References list and also include a citation and full reference for the retraction notice. | We have reviewed the reference list and it is complete and correct. In response to reviewer #4’s comments, we added two citations to our revised manuscript:   - Fuhrmeister, Erica R., et al. "Predictors of enteric pathogens in the domestic environment from human and animal sources in rural Bangladesh." *Environmental science & technology* 53.17 (2019): 10023-10033. - Bauza, Valerie, et al. "Enteric pathogens from water, hands, surface, soil, drainage ditch, and stream exposure points in a low-income neighborhood of Nairobi, Kenya." *Science of the total environment* 709 (2020): 135344. |
| **Reviewers’ comments** | |
| R3.1. I am fully satisfied with this corrected version of this article. I respect authors' right to do not respond to some minor recommendations. | We appreciate the reviewer’s comments and thank them for their time. |
| R4.1. Thank you for your revisions and incorporating my comments. However, please address the following comment in the discussion section and please refer to my previous comment in the previous revision for context.  The discussion needs incorporate findings of other papers in the literature in greater detail. This includes comparing and contrasting the authors' findings with other studies which have contrasting results.  For example, in lines 272 of tracked version, the authors note that “handwashing after removing feces was not always mentioned and surfaces were inconsistently cleaned with soap or disinfectants”. This is an important point to address considering other studies note that handwashing can lower infant exposure to pathogens. Other points that the authors could discuss include their results from Table 1 such as latrine features, floor material etc. Studies in the literature have identified these features to be associated with infant pathogen exposure.  These are just a few examples of how the authors can enhance their discussion. Please add additional examples of discussing results with other papers in the literature. | We thank the reviewer for additional clarity and the opportunity to incorporate their suggestion. We have added more detailed discussion of literature, where appropriate, including the following:   - Although 66% of households had access to improved drinking water sources and 81% had improved sanitation facilities, all mothers reported opportunities for their child to be exposed to animal feces, even though 45% of households did not own animals. These findings are in line with other studies showing that WASH interventions aiming to reduce enteric infections by targeting human feces alone likely overlook other significant sources of environmental fecal contamination and enteric pathogens. The findings also highlight that focusing only on animal ownership and only on household environments provide insufficient information to identify the relative risk of child exposure to enteric pathogens in animal feces. [Lines 354-359] - Existing evidence clearly demonstrates that child household and play spaces can be contaminated with animal feces and associated enteric pathogens when free-range animals are present, and that proximity to animals and such contaminants increases the risk of enteric infections among children. Our findings add to existing research by highlighting that child exposure to enteric pathogens in animal feces is not solely shaped by household practices but is also significantly shaped by the normative practices of others in their community. Findings further suggest that sole focus on the household environment provides inaccurate and/or incomplete data because other significant locations where children may be exposed to enteric pathogens in animal feces can be missed. Consequently, research and interventions exclusively targeting the household level may be insufficient to examine and reduce child exposure to animal feces. [Lines 365-375] - While animal feces near child domestic and play areas were removed the majority of the time, maternal handwashing was seldom discussed and surfaces were inconsistently cleaned with soap or disinfectants after removal of animal feces. In Bangladesh, maternal hand contamination with animal feces was near universal despite the reported use of tools to clean up animal feces and in rural India, removing child feces without using tools was associated with increased hand contamination. Findings from these studies suggest that handwashing after handling animal feces could help reduce child exposure to enteric pathogens, especially given studies showing strong correlations between animal feces and enteric pathogen contamination on caregiver and child hands in the same household. Similarly, cleaning or disinfecting surfaces after removal could reduce exposure, as a study in rural India found that environmental fecal contamination remains even after child feces are removed. [Lines 377-387]   As written, we now position our findings among 24 papers, four of which are reviews and three of which discuss 2+ studies. |
| R5.1. The authors are commended for having done a rigorous job in addressing comments made by reviewers. For reviewer comment R5.2, though the authors responded that animal health is outside their scope, they have responded accordingly in lines 37-38.  I have a few areas I have noted minor issues in wording as follows:  1. Line 211- Change "of" to "from"  2. Line 218: Change "child" to "child's"  3. Line 262: Remove the word "of"  4. Line 271: Change "animal" to "animals" | We appreciate the reviewer’s comments and have made the four minor changes. |
